# Tinder Users: Sociodemographic, Psychological, and Psychosexual Characteristics

**DOI:** 10.3390/ijerph17218047

**Published:** 2020-10-31

**Authors:** Juan Ramón Barrada, Ángel Castro

**Affiliations:** Facultad de Ciencias Sociales y Humanas, Universidad de Zaragoza, Calle Atarazanas 4, 44003 Teruel, Spain; barrada@unizar.es

**Keywords:** dating apps, Tinder, Tinder motives scale, psychosexual variables, university students

## Abstract

Tinder is the most popular and most used dating app in the world today. Due to the recent popularization of the phenomenon of dating apps, there are still some gaps in the literature. Therefore, this study had a threefold objective: (1) to determine the prevalence and characteristics of Tinder users and Tinder use among young university students; (2) to know why these young people use Tinder; and (3) to analyze the relationship between Tinder use and different psychosocial correlates (positive and negative affect, body satisfaction, sociosexuality, and attitudes towards consensual nonmonogamy) and psychosexual well-being (self-esteem as a sexual partner, satisfaction with sex life, and preoccupation with sex). Participants were 1261 Spanish university students (77.4% women, 77.5% heterosexuals) between ages 18 and 26 (*M* = 20.59, *SD* = 2.04) who completed a battery of online questionnaires. A prevalence of Tinder use of about 15% was found. The motives for use most frequently reported by the participants were those of curiosity, passing time/entertainment, and sexual orientation. Besides, Tinder users showed greater sociosexuality than nonusers, as well as increased dissatisfaction with their sex life and sexual preoccupation, and more positive attitudes towards consensual nonmonogamy. Importantly, no or very small differences were found in the general emotional well-being-related variables. Tinder seems like just another tool used by young people for their romantic and/or sexual interactions, without any negative connotation.

## 1. Introduction

The development of online dating has grown considerably in recent years and has changed the way some people interact with potential new romantic and/or sexual partners, especially after the emergence and popularization of dating apps [1]. In fact, different studies have shown that around 40% of single adults are looking for an online partner [2] or that about 25% of new couples met each other through this means [3].

The real-time location-based dating apps (e.g., Tinder and Grindr), with a very simple and intuitive functioning (see the description of how Tinder works, for example, in [4]), have great advantages over other forms of dating, including sites and websites [5]. Some authors, such as Ranzini and Lutz [6] or Timmermans and De Caluwé [7], highlighted four advantages of these applications based on the technology they use and the possibilities they offer. First, mobility, which allows the use of the apps in different places, both public and private. Second, immediacy, as notifications and alerts accelerate the time of interactions and allow a greater spontaneity and directness. Thirdly, proximity, as potential couples who are geographically close to the user emerge. Finally, the visual aspect, because they are specially oriented towards physical appearance, through photos and a brief self-description.

Tinder, created in 2012, is the most popular and frequently used dating app in the world [5]. It is estimated to be present in nearly 200 countries and has more than 50 million active users, with around 10 million daily users [5,8,9]. In recent years, in parallel to the development and growing popularity of Tinder, the scientific literature on this phenomenon has increased, so studies on usage prevalence and user profile, motives for use, and their relationship with different kinds of variables can be found, as reported by Castro and Barrada [10] in their systematic review.

### 1.1. Characteristics of Tinder Users and Tinder Use

It is difficult to know the prevalence of Tinder use, as the data vary depending on the context of the studies, the groups evaluated, and the sampling technique used. In fact, some studies conclude that between 40% and 50% of young people use some dating app [11,12,13], but the results of other studies with a lower sample bias reduce that prevalence. For example, in their study with Norwegian university students, Botnen et al. [14] found that 20% were current users. Timmermans and De Caluwé [7] found a 22% prevalence of use among Belgian university students. Castro et al. [15] found that 15.8% of the Spanish university students who participated in their study were previous users and 12.7% had used dating apps in the last three months. This disparity in approaches and data has led some authors to criticize the over-representation of dating app users reported in some studies, making it difficult for researchers to know the reality [10].

As for users’ sociodemographic characteristics, in their study with Spanish university students, Castro et al. [15] confirmed results from previous studies conducted in other contexts and concluded that men [13,16], older youths (in the range 18–26 years) [17], single people [18], and members of sexual minorities [19,20] presented higher rates of dating app use.

Tinder users are estimated to check the app an average of 11 times a day, investing up to 90 min a day [5], although these results differ depending on sex and sexual orientation, with men and people from sexual minorities being those who use the apps with the most frequency [18,19].

There is considerable variability in the results related to the offline behavior of Tinder users. For example, it has been found that between 33% [11] and 70% [4] of users have had a date with someone they met on Tinder, with an average of 4.25 dates per user [7]. According to Strugo and Muise [4], 51% of users have had sex with someone they met on Tinder, with an average of 1.57 partners [7]. In addition, between 17% [18] and 33% [4] have had some romantic relationship with someone met on Tinder. Finally, Timmermans and De Caluwé [7] found an average of 2.19 friendship relationships emerging between Tinder users.

### 1.2. Motives for Tinder Use

Since its inception, there is a belief that Tinder is a “hook-up app”, which is mainly used to have casual sex [4]. However, the existing literature refutes this stereotype [10]. For example, Sumter and Vandenbosch [13] referred to young people using Tinder both for relational (e.g., looking for a partner for casual sex or a romantic relationship), intrapersonal (e.g., ease of communication and ego enhancement), and entertainment reasons (e.g., curiosity and trendiness). Thus, it seems that Tinder can serve different purposes, among which, the search for casual sex is only one [6,8,21,22].

In recent years, as a result of the expansion of the use of Tinder, instruments and classifications of motives for its use have proliferated. The first available one is that of Ranzini and Lutz [6], who identified six motivations (hooking-up/sex, friendship, relationship, traveling, self-validation, and entertainment). Subsequently, Sumter et al. [21] and Sumter and Vandenbosch [13] developed a classification of six motives for Tinder use (love, casual sex, ease of communication, self-worth validation, thrill of excitement, and trendiness). Orosz et al. [8] validated the Tinder use motivations scale, composed of four groups of motives (boredom, self-esteem, sex, and love).

However, the most complete and comprehensive tool for evaluating the motivations for Tinder use is the Tinder motives scale (TMS) [22]. This scale, developed and validated with Belgian Tinder users, consists of 58 items, grouped into 13 factors/motives, with adequate levels of reliability (between 0.70 and 0.95). All items are rated on a seven-point response scale, which ranges from 1 = *strongly disagree* to 7 = *strongly agree*. The 13 motives evaluated by the scale are: social approval (six items), relationship-seeking (five items), sexual experience (six items), flirting/social skills (six items), traveling (five items), ex (three items), belongingness (four items), peer pressure (three items), socializing (four items), sexual orientation (three items), passing time/entertainment (seven items), distraction (three items), and curiosity (three items).

The main motives for Tinder use found by Timmermans and De Caluwé [22] were passing time/entertainment (*M* = 5.02), curiosity (*M* = 4.49), and socializing (*M* = 4.21), thus showing that young people use Tinder for various motives and not so much for the mere search for casual sex. Concerning the TMS, its authors emphasized the adequate reliability and validity of the scale but suggested that it would be important to assess its usefulness and adequacy in other geographical and cultural contexts [22].

The existing literature on motives for using Tinder shows that they vary depending on gender, age, and sexual orientation [10,17]. Men and people from sexual minorities use Tinder more to have casual sex, while women and heterosexuals do so in a greater proportion for other reasons [9]. Additionally, it seems that motivations change as people grow and mature, with direct relationships between age and the search for relationships, both casual and romantic, and inverse relationships with the motives of peer-pressure and self-validation [6,8,21].

### 1.3. Correlates of Tinder Use

The study of the relationship between Tinder use and certain personality traits (e.g., Big Five and Dark Tetrad) was inconclusive. For example, Castro et al. [15] found that the only trait that could predict the current use of dating apps was open-mindedness. Timmermans and De Caluwé [7] examined the use of dating apps, these personality traits, and the relational status, finding that single users of Tinder were more outgoing and open to new experiences than nonusers, who scored higher in conscientiousness. Therefore, the scarce existing literature can lead to the conclusion that the role of personality in the use of dating apps is unclear.

Tinder, being an app focused almost exclusively on appearance, can encourage the use of patterns of behavior and functioning that relate to physical, mental, and relational health, as well as to its users’ well-being [17]. Besides, due to the recency of the phenomenon, the existing research is not very broad and has focused on general, and not on very specific, variables. For example, the problematic use of Tinder was found to have adverse psychological effects, such as a greater sense of loneliness and dissatisfaction with life [23,24]. However, the relationship between the moderate and recreational use of Tinder and mood, which could provide relevant information for researchers and clinicians, has not been studied.

The same goes for psychosexual well-being [1]. Although self-esteem as a predictor and correlate of Tinder use has been studied, although with inconclusive results [10], it was done with general self-esteem. It would be interesting to analyze this relationship with sexual self-esteem and expand it with the inclusion of other well-being variables, such as satisfaction with sex life or sexual preoccupation.

The relationship between Tinder use and body satisfaction has been studied. The relevance of physical appearance in dating apps can promote excessive concerns about body image, as well as different negative consequences associated with that concern (e.g., unhealthy weight, weight-management behaviors, and low body satisfaction; see [25,26]).

Sociosexuality is another variable related to the use of Tinder [1]. It was found that users of this app, as seems logical, tend to have less restrictive sociosexuality, especially those who use it to have casual sex [2,14]. In this line, and given the extension of consensual nonmonogamy as an expanding relational model [27], it would be interesting to take it a step further and determine the relationship between being a Tinder user and this affective-sexual option.

### 1.4. The Present Study

Despite its current relevance and growing importance in human relations, Tinder remains a novel phenomenon, requiring more research. With this study, we wished to meet a triple goal. On the one hand, we wanted to determine the prevalence and sociodemographic characteristics of Tinder users and Tinder use in a sample of young Spanish university students. Secondly, we wanted to know why these young people use Tinder, which will facilitate the comparison of the results with those of studies carried out in other countries (e.g., Belgium, The Netherlands, and Hungary) and allow us to evaluate the image of this dating app as a space oriented towards casual sex. Thirdly, we wished to analyze the relationship between Tinder use and different psychosocial correlates (positive and negative affect, body satisfaction, sociosexuality, and attitudes towards consensual nonmonogamy) and psychosexual well-being (self-esteem as a sexual partner, satisfaction with sex life, and sexual preoccupation). This information, in addition to being novel, can have implications for the work of researchers, clinicians, and prevention professionals because of the relevance that dating apps are acquiring nowadays and their relationship with physical, mental, and relational health.

## 2. Materials and Methods

### 2.1. Participants and Procedure

The initial sample comprised 1562 participants. Four inclusion criteria were used: (1) to be studying a university degree at the time of data collection (129 participants excluded), (2) to be aged between 18 to 26 years, according to criteria from previous studies with university samples (120 participants excluded) [15,28,29], (3) to label oneself as a woman or a man (12 participants excluded; the small sample size of this group prevented us from incorporating these participants into our analysis), and (4) to correctly answer a control question (40 participants excluded; see below).

After applying these criteria, the final sample included 1261 university students (77.4% women and 22.6% men), aged between 18 and 26 (*M* = 20.59, *SD* = 2.04). Of these participants, 77.5% described themselves as heterosexual, 15.8% as bisexual, 5.1% as homosexual, and 1.7% as other orientations. Due to the small sample sizes of nonheterosexual groups, those participants were combined into a sexual minority category (22.5%).

Regarding the procedure, data were collected in December 2017 and January 2018 using a Google Forms survey. To reach participants, a link to the survey was distributed through the e-mail distribution lists of the students of the authors’ university. The survey remained open for 30 days. Participants provided informed consent after reading the description of the study, where the anonymity of the responses was clearly stated. This procedure was approved by the Ethics Review Board for Clinical Research of the region (PI18/058).

### 2.2. Measures

#### 2.2.1. Sociodemographic and Tinder Use Questionnaire

We asked participants about their gender (woman, man, or other), age, and sexual orientation (heterosexual, homosexual, bisexual, or other). We asked participants if they had used or were using Tinder. Those who answered “yes” were asked for how long (in months) they had been active users; their frequency of use; and the number of people met on Tinder with whom they have had: (1) a face-to-face meeting; (2) sex (oral, vaginal, and/or anal); (3) a romantic relationship; and (4) a friendship.

#### 2.2.2. Tinder Motives Scale

Only those participants who were current or previous users of Tinder responded to this questionnaire. This instrument (TMS) [22] has 58 items that assess the motives for using Tinder on the basis of 13 dimensions: passing time/entertainment (e.g., [I use Tinder...] “to pass time”; α = 0.85—all reported alphas correspond to values obtained with the current sample), curiosity (e.g., “out of curiosity”; α = 0.80), socializing (e.g., “to meet new people”; α = 0.68), relationship-seeking (e.g., “to find someone for a serious relationship”; α = 0.91), social approval (“to get compliments”; α = 0.87), sexual orientation (e.g., “to get to know people with the same sexual orientation”; α = 0.91), flirting/social skills (e.g., “to learn to flirt”; α = 0.84), distraction (e.g., “to combat boredom when working or studying”; α = 0.73), sexual experience (e.g., “to increase my sexual experience”; α = 0.88), traveling (e.g., “to meet other travelers/locals when in a foreign country”; α = 0.94), peer pressure (e.g., “because my friends thought I should use Tinder”; α = 0.74), ex (“to get over my ex”; α = 0.93), and belongingness (e.g., “because I want to be trendy”; α = 0.85). It is rated on a seven-point Likert type scale ranging from 1 = *strongly disagree* to 7 = *strongly agree*.

#### 2.2.3. Short Version of the Sexuality Scale

This instrument (Short version of the Sexuality Scale, SSS) [30,31] has 15 items that assess the perceptions of one’s sexuality through three components: self-esteem as a sexual partner (e.g., “I am a good sexual partner”; α = 0.89), dissatisfaction with sexual life (e.g., “I’m depressed about the sexual aspects of my life”; α = 0.92), and sexual preoccupation (e.g., “I’m constantly thinking about having sex”; α = 0.90). The items are rated on a five-point Likert-type scale ranging from 1 = *strongly disagree* to 5 = *strongly agree*. We used the Spanish adaptation of Soler et al. [32].

#### 2.2.4. Positive and Negative Affect Schedule

The Positive and Negative Affect Schedule (PANAS) [33] has 20 items measuring both positive and negative affect, with 10 items per dimension. Participants are asked to rate on a five-point Likert scale, from 1 = *very slightly or not at all* to 5 = *extremely*, how much they experience different feelings and emotions, such as “enthusiastic” (α = 0.86) for a positive affect or “nervous” (α = 0.85) for a negative affect. We used the Spanish adaptation of Sandin et al. [34].

#### 2.2.5. Appearance Evaluation Scale of the Multidimensional Body-Self Relations Questionnaire-Appearance Scales

This instrument (MBSRQ) [35] assesses the degree of satisfaction with one’s body. It is composed of seven items (e.g., “I like the way my clothes fit me”; α = 0.91), with a five-point response scale ranging from 1 = *definitely disagree* to 5 = *definitely agree*. We used the Spanish adaptation of Roncero et al. [36].

#### 2.2.6. Sociosexual Orientation Inventory-Revised

This instrument (SOI-R) [37] has nine items that assess sociosexual orientation on the basis of three dimensions: behavior (e.g., “With how many different partners have you had sexual intercourse without having any interest in a long-term committed relationship with this person?”; α = 0.93), attitudes (e.g., “Sex without love is OK”; α = 0.81), and desire (e.g., “How often do you have fantasies about having sex with someone with whom you do not have a committed romantic relationship?”; α = 0.91). These items are rated on a nine-point scale, ranging from 1 = *0* to 9 = *20 or more* in the behavior factor, from 1 = *strongly disagree* to 9 = *strongly agree* in the attitudes factor, and from 1 = *never* to 9 = *at least once a day* in the desire factor. We used the Spanish validation of Barrada et al. [38].

#### 2.2.7. Consensual Nonmonogamy Attitude Scale

This instrument (CNAS) [39] has eight items to determine how accepting people are of consensual nonmonogamy attitudes (e.g., “I can see myself entering into a non-monogamous relationship”; α = 0.80). The items are rated on a seven-point Likert-type scale ranging from 1 = *strongly disagree* to 7 = *strongly agree*.

#### 2.2.8. Control Question

Embedded in the SSS as its sixteenth item and to check whether the participants paid enough attention to the wording of the items, we introduced an item asking the participants to respond to it with *strongly disagree*. Participants responding with a different option from the one requested could be considered distracted.

#### 2.2.9. Translation and Adaptation of the TMS and CNAS

The English version of the TMS and the CNAS were translated into Spanish by two experts in sexuality research using a forward translation procedure. Both the translated and the original versions were given to a bilingual expert in translating psychological and sexological manuscripts to ensure the correspondence between the two versions. Then, the Spanish translations were analyzed by two experts in psychological assessment and sexuality research to identify and suggest changes to items that were not clear and understandable. No changes were made at this phase of the study. Finally, the resulting versions were given to two individuals with characteristics similar to the final sample. They received the same task as the experts in psychological assessment and sexuality research. No changes were made at this phase, either. The Spanish version of both the TMS (Table A1) and CNAS (Table A2) are included as appendices.

### 2.3. Data Analyses

Firstly, we compared Tinder users and nonusers in three sociodemographic variables (age, gender, and sexual orientation) and the ten psychosexual and psychological measured variables. The association between the Tinder use group and the numerical variables was quantified with Cohen’s *d*, and for dichotomous variables, it was quantified with Cramer’s *V*. Secondly, we computed a logistic regression model, with the Tinder use group as the criteria and sociodemographic information as the predictors.

Thirdly, we computed a multiple linear regression analysis of the Tinder use characteristics. We predicted the usage time, use frequency, number of people met, number of sexual relationships with Tinder contacts, number of romantic relationships, and number of friendships by means of sociodemographic information.

Fourthly, a similar approach was followed to predict the scores in the TMS. As the metric of these questionnaire scores is not easy to interpret, we standardized them before the regression. By doing so, the *b* coefficients of the predictors indicated the expected change in each of the TMS scores for increments of a year of age or differences between men and women and between heterosexual and sexual minority participants.

Fifthly, we estimated the multiple linear regression models for the 10 different psychological and psychosexual variables. The predictors were the three sociodemographic variables plus the Tinder use group. Again, the criteria variable scores were standardized. The *b* coefficients had the same interpretations as for the previous models, and for the Tinder use variable, they represented the differences between users and nonusers.

The analyses were performed with R 4.0.3 (R Foundation for Statistical Computing, Vienna, Austria) [40]. The open database and code files for these analyses are available at the Open Science Framework repository (https://osf.io/27m3x/).

## 3. Results

The associations among Tinder use and the sociodemographic, psychological, and psychosexual information can be seen in Table 1. Of the participants, 86.0% (*n* = 1085) were Tinder nonusers and 14.0% (*n* = 176) were users. All sociodemographic variables were associated with the dating apps users group. With respect to gender, for women, the distributions by group were *p*_nonuser_ = 0.87 and *p*_user_ = 0.13; for men, *p*_nonuser_ = 0.81 and *p*_user_ = 0.19; χ^2^(1) = 6.60, *p* = 0.010, *V* = 0.07. For sexual minority participants, *p*_nonuser_ = 0.75 and *p*_user_ = 0.25; for heterosexual participants, *p*_nonuser_ = 0.89 and *p*_user_ = 0.11; χ^2^(1) = 39.63, *p* < 0.001, *V* = 0.18. Age was associated with the Tinder users group, with users being the older ones (*M* = 21.40, *SD* = 2.03) and nonusers the younger (*M* = 20.46, *SD* = 2.01), *t*(1259) = 5.72, *p* < 0.001, *d* = 0.46.

Tinder users and nonusers showed statistically significant differences in all psychosexual and psychological variables but not in body satisfaction [*t*(1259) = −0.59, *p* = 0.557, *d* = −0.05] and self-esteem as a sexual partner [*t*(1259) = 1.45, *p* = 0.148, *d* = 0.12]. Differences in both negative [*t*(1259) = 1.96, *p* = 0.050] and positive affects [*t*(1259) = 1.99, *p* = 0.047] were rather small, *d*s = 0.16. Tinder users presented higher dissatisfaction with sexual life [*t*(1259) = 3.73, *p* < 0.001, *d* = 0.30]; preoccupation with sex [*t*(1259) = 4.87, *p* < 0.001, *d* = 0.40]; and better attitudes to consensual nonmonogamy [*t*(1259) = 4.68, *p* < 0.001, *d* = 0.38]. The larger differences were in the three sociosexual dimensions [behavior, *t*(1259) = 10.20, *p* < 0.001, *d* = 0.83; attitudes, *t*(1259) = 5.30, *p* < 0.001, *d* = 0.43; and desire, *t*(1259) = 8.06, *p* < 0.001, *d* = 0.66], with Tinder users more oriented toward short-term relationships.

Results of the logistic regression model are shown in Table 2 and were in accordance with those just reported. For this model, the explanatory capacity was small (Nagelkerke’s pseudo-*R*^2^ = 0.10 and McFadden’s pseudo-*R*^2^ = 0.07). Men had a higher probability of Tinder use (odds ratio, OR = 1.52, *p* = 0.025). Increments in age were associated with increments in the probability of use (OR = 1.25, *p* < 0.001). Being heterosexual reduced the probability of use (OR = 0.35, *p* < 0.001). To better understand the relevance of these variables, we computed the probability of Tinder use for an 18-year-old heterosexual woman and for a 26-year-old nonheterosexual man. For that woman, *p*_user_ = 0.05; for that man, *p*_user_ = 0.59.

Results of the regression models for Tinder use characteristics and their descriptives are shown in Table 3. Tinder users had been using the app for 4.04 months and 10.14 times per week. Users met a mean of 2.59 Tinder contacts offline and had 1.32 sexual relationships. As the average, the use of the app led to 0.27 romantic relationships and 0.85 friendships.

For the six considered characteristics, five regression models showed significant results with *p*s ≤ 0.036 (all but the number of romantic relationships, *p* = 0.253), but all the $R_{adj}^{2}$ were small (range [0.01, 0.10]). Given the large number of estimated coefficients, we restricted our attention to those statistically significant. Men tended to use Tinder for a longer time (*b* = 2.14, *p* = 0.032) and gained more friends via Tinder (*b* = 0.70, *p* = 0.008). Sexual minority participants met a larger number of people offline (*b* = −1.33, *p* = 0.029), had more sexual relationships (*b* = −0.98, *p* = 0.026), and gained more friends via Tinder (*b* = −0.81, *p* = 0.001). Older participants used Tinder for longer (*b* = 0.51, *p* = 0.025), with more frequency (*b* = 0.72, *p* = 0.011), and met more people (*b* = 0.30, *p* = 0.040).

Results of the regression models for Tinder motives and their descriptives are shown in Table 4. The results were ordered in descending order by the score means. The motives with higher means were curiosity (*M* = 4.83; response scale 1–7), pastime (*M* = 4.44), and sexual orientation (*M* = 4.15). Those with lower means were peer pressure (*M* = 2.20), ex (*M* = 2.17), and belongingness (*M* = 1.66).

For the 13 considered motives, seven regression models showed significant results (*p*s ≤ 0.038), and six were statistically nonsignificant (*p*s ≥ 0.077). The $R_{adj}^{2}$ tended to be small (range [0.00, 0.13]). Again, we only commented on those statistically significant coefficients (when the overall model was also significant). Women reported higher scores for curiosity (*b* = −0.53, *p* = 0.001), pastime/entertainment (*b* = −0.46, *p* = 0.006), distraction (*b* = −0.38, *p* = 0.023), and peer pressure (*b* = −0.47, *p* = 0.004). For no motive men’s means were higher than women’s. While sexual minority participants showed higher scores for sexual orientation (as could be expected; *b* = –0.75, *p* < 0.001) and traveling (*b* = −0.37, *p* = 0.018), heterosexual participants had higher scores for peer pressure (*b* = 0.36, *p* = 0.017). Older participants tended to be more motivated by relationship-seeking (*b* = 0.11, *p* = 0.005), traveling (*b* = 0.08, *p* = 0.035), and social approval (*b* = 0.08, *p* = 0.040).

The results for the 10 psychological and psychosexual variables are shown in Table 5. All the regression models were statistically significant (all *p*s < 0.001). Again, the $R_{adj}^{2}$ tended to be small, with $R_{adj}^{2}$ in the range [0.01, 0.15]. Given the focus of the manuscript, we only described the differences according to Tinder use. The other coefficients were less informative, as they corresponded to the effects adjusted for Tinder use. Importantly, Tinder users and nonusers did not present statistically significant differences in negative affect (*b* = 0.12, *p* = 0.146), positive affect (*b* = 0.13, *p* = 0.113), body satisfaction (*b* = −0.08, *p* = 0.346), or self-esteem as a sexual partner (*b* = 0.09, *p* = 0.300), which are the four variables related to the more general evaluation of the self. Tinder users showed higher dissatisfaction with sexual life (*b* = 0.28, *p* < 0.001), a higher preoccupation with sex (*b* = 0.37, *p* < 0.001), more sociosexual behavior (*b* = 0.65, *p* < 0.001), a more positive attitude towards casual sex (*b* = 0.37, *p* < 0.001), a higher sociosexual desire (*b* = 0.52, *p* < 0.001), and a more positive attitude towards consensual nonmonogamy (*b* = 0.22, *p* = 0.005).

## 4. Discussion

Tinder is the most popular and frequently used dating app in the world, present in almost 200 countries and with more than 50 million active users [5]. Its emergence and increasing popularization have contributed decisively to changes in the ways and forms of interaction with potential partners that have taken place in recent years. However, due to the recency of the phenomenon, the existing literature on Tinder presents some gaps. Therefore, the objective of this study was threefold. On the one hand, we wanted to determine the prevalence and characteristics of Tinder users and Tinder use in a sample of young Spanish university students, and, on the other hand, to know why these young people use Tinder, and, finally, to analyze the relationship between Tinder use and different psychosocial and psychosexual well-being correlates.

Once the three objectives of the study were met, different conclusions could be drawn, and several aspects were proposed for discussion. First, concerning Tinder use and Tinder users, a prevalence of use of about 15%, similar to that found by Castro et al. [15], was found in a different sample of Spanish university students. It should be noted that this result may be influenced by some methodological issues. On the one hand, the questionnaire asked whether Tinder had been used or was being used, taking into account both previous and current users, which may over-represent the prevalence of use. However, in contrast, given that Tinder users had to answer 58 more items (belonging to the TMS), some users who did not complete the questionnaire were probably lost, leading to an infra-estimation of lifetime Tinder users.

In any case, the prevalence of Tinder use found was lower than, although close to, that of other studies conducted with university students [7,14], but it was far from the prevalence of 40% to 50% found by other researchers [11,12,13]. These great differences force one to insist on the risk that Castro and Barrada [10] warned about in their systematic review. The studies use different methods of sampling and data collection, and, in some cases, this methodology tends to overestimate the users. This makes it difficult for researchers to work, and it can distort their results and decision-making.

The usage data found in Spanish participants were also lower than in previous studies. Both the frequency of use [5] and the number of people who met on Tinder with whom they had a date or a romantic or friendship relationship [4,7,11] were lower. The only similar result was the number of people met on Tinder with whom they had sex: 1.32 in this study and about 1.57 in the work of Timmermans and De Caluwé [7].

There are two possible explanations for the differences found in the prevalence and intensity of use between young Spaniards and participants in previous studies. The first is cultural. In Spain, although it is clear that Tinder is a well-known and frequently used tool, other forms of dating, more face-to-face and associated with climate, culture, and lifestyles, may still be very strong. This could mean that, in proportional terms, fewer people use Tinder, and this is done with less intensity than in other cultural contexts. Secondly, these differences can also be explained by the participants’ ages. Other research used wider age ranges, also among college students, reaching age 30. As some studies have concluded that there is a direct relationship between age and the use of dating apps [15,17], the prevalence and intensity of use are likely to be higher as participants approach 30 years of age.

It was also found that young Spanish university students used Tinder for similar reasons as young people in other countries. The two main motivations for using Tinder were curiosity and passing time/entertainment, which were the same, albeit in a different order, as in the study of Timmermans and De Caluwé [22]. Coincidence also were the two motives less frequently pointed out by the participants, those that have to do with the ex-partner and belongingness.

It seems clear, on the one hand, that TMS is an exhaustive tool for assessing Tinder’s motivations for use. On the other hand, young people use Tinder mainly out of curiosity, entertainment, and to socialize, so we should banish the idea that Tinder is a “hook-up app”, associated with superficiality and sexual frivolity [8,10,21,22,41].

Differences were found in the motives for use depending on sociodemographic variables (gender, age, and sexual orientation). It is relevant to highlight the differences found based on gender. While women reported using Tinder to a larger degree due to curiosity, pastime, distraction, or peer pressure, men showed higher in any of the motives. In opposition to Sevi et al. [9], men did not used Tinder to have casual sex and gain sexual experience to a greater extent than women. These results, which should be confirmed in future studies, are in-line with the fact that women in Spain are increasingly owners of their sexuality, and they decide not only when and with whom they have sex, but they also feel free to experience and satisfy their entertainment and curiosity.

One of the main contributions of this study is the inclusion of psychosocial and psychosexual variables associated with well-being, as well as the comparisons between Tinder users and nonusers in these variables. As for the former, no differences were found between Tinder users and nonusers in mood (positive and negative affect) or body satisfaction, which previous studies did find [23,25,26]. The higher differences between Tinder users and nonusers were in the sociosexuality (behavior, attitudes, and desire) and attitudes towards consensual nonmonogamy. In this study, as in the study of Sevi et al. [9], users of Tinder— an app with some orientation toward casual sex—were expected to score higher in sociosexuality and be more open to different types of relationships and partners than nonusers.

Given that no differences in self-esteem as a sexual partner were found, the only differences that can be associated with psychosexual well-being are those that have to do with dissatisfaction with sex life and sexual preoccupation. Tinder users rated higher on both. In future studies, the causality between these variables should be studied; whether dissatisfaction with sex life and sexual preoccupation motivate the use of Tinder or whether, on the contrary, being on Tinder and the functioning of the app and the types of relationships it generates motivate that discomfort. It may also be simply that the most erotophilic people and/or those who want more sex are on Tinder.

In any case, the results obtained concerning mood and affect suggest that being a Tinder user is not associated with negative aspects in these areas beyond some punctual aspects (e.g., higher sexual preoccupation and sexual dissatisfaction) that should be further investigated. It seems that this app is just another tool that young people have to interact with and relate to each other, without negative connotations.

The study has a number of limitations that need to be taken into account when interpreting the results. First, some aspects related to sample selection should be discussed. As was already stated, and as in prior studies [13,26], both previous and current users of Tinder were taken into account, not distinguishing between the two, which can generate some overestimation of the prevalence of use. In contrast, some Tinder users may not have finished completing their questionnaire, which was more extensive than that of nonusers; therefore, their data were lost. In addition, by not distinguishing between previous and current users, it was not very useful to know whether the participants currently had a romantic partner, so no differences were evaluated based on relationship status, a relevant variable in other studies, as reported by Castro and Barrada [10].

As for the final sample of participants, it was mostly female, between the ages of 18 and 26 and from a single university, so the results are difficult to generalize to university students and, still less, to non-university youths. Similarly, when deciding to group participants into heterosexuals and nonheterosexuals, we lost information about the specific characteristics of Tinder use and its motives and correlations among members of sexual minorities. In addition, our study shares with other studies based on self-selected samples and self-reported measures the fact that the results may be limited by response and recall bias. Finally, a limitation that is shared with the existing literature on the subject is that studies are cross-sectional. It would be interesting to carry out longitudinal studies in which the evolution in the uses and motives of users, as well as their correlates, could be evaluated.

Despite these limitations, the study makes relevant contributions, offering data on Tinder users and Tinder use in a geographical and cultural context that has been very little studied to this day, such as Spain. Information was provided on why young Spanish university students use Tinder, thus allowing comparison with the results of studies carried out in other contexts. Novel information was provided on the use of Tinder and its relationship to psychosocial and psychosexual well-being.

## 5. Conclusions

The growing popularity of Tinder, as well as of other dating apps, raises a number of questions to answer. One of them is about the mental health and emotional well-being of users compared to those of nonusers. With this study, it was shown that, despite some higher level of dissatisfaction and sexual preoccupation, participants using Tinder did not present worse moods, less body satisfaction, or lower self-esteem as a sexual partner than nonusers.

Therefore, these results suggest that Tinder is just another tool, increasingly known and used by young people to contact and interact with potential romantic and/or sexual partners, and that young people use it normally and behave on it similarly as they do in real life. At least, for them, it has no negative connotations, nor does its use imply the loss of emotional well-being.

This could have several implications, both from theoretical and the applied approaches. At the theoretical level, we think that the findings of the study increase our knowledge about the psychological and psychosexual correlates of Tinder use. These results serve to combat some stereotypes about the use of this kind of apps (e.g., users have a worse mood) and can be a starting point for further research into the role of health and psychological and psychosexual well-being in the use of Tinder and other dating apps.

At the applied level, the results of the study may have notable implications for several groups. First, for clinicians and professionals of the prevention and promotion of mental, sexual, and relational health, who will have more tools to deal with topics as novel as those related to dating apps. Second, for the users themselves and for potential users, as well as for society at large, because this study can serve to demystify the somewhat the negative connotation that dating apps still currently have in some contexts.

## Figures and Tables

**Table 1:** Means and standard deviations for age, proportions for gender and sexual orientation, and significance testing according to Tinder use.

	Nonusers	Users			
	**Proportion**		**χ^2^**	***p***	***V***
Gender			6.60	0.010	0.07
Women	0.87	0.13			
Men	0.81	0.19			
Sexual orientation			39.63	<0.001	0.18
Sexual minority	0.75	0.25			
Heterosexual	0.89	0.11			
	**Mean (Standard Deviation)**		***t***	***p***	***d***
Age	20.46 (2.01)	21.40 (2.03)	5.72	<0.001	0.46
PANAS Negative	21.80 (6.71)	22.89 (7.47)	1.96	0.050	0.16
PANAS Positive	34.49 (6.52)	35.53 (5.86)	1.99	0.047	0.16
MBSRQ	25.01 (6.35)	24.71 (6.53)	−0.59	0.557	−0.05
SSS Sexual Partner	18.80 (4.01)	19.28 (4.20)	1.45	0.148	0.12
SSS Dissatisfaction	9.69 (5.10)	11.25 (5.49)	3.73	<0.001	0.30
SSS Preoccupation	8.52 (4.13)	10.20 (5.01)	4.87	<0.001	0.40
SOI-R Behavior	7.84 (5.27)	12.43 (6.95)	10.20	<0.001	0.83
SOI-R Attitude	17.48 (6.80)	20.35 (5.85)	5.30	<0.001	0.43
SOI-R Desire	9.78 (5.30)	13.28 (5.60)	8.06	<0.001	0.66
CNAS	29.42 (10.19)	33.35 (11.05)	4.68	<0.001	0.38

Nonusers: participants reported having never used Tinder. Users: participants reported having ever used Tinder. *d* = Cohen’s *d*. *V* = Cramer’s *V* Age, measured in years. Proportions by row. PANAS = Positive and Negative Affect Schedule. MBSRQ = Appearance Evaluation Scale of the Multidimensional Body-Self Relations Questionnaire-Appearance Scales. SSS = Short version of the Sexuality Scale. SOI-R = Sociosexual Orientation Inventory-Revised. CNAS = Consensual Nonmonogamy Attitude Scale. Sexual Partner = self-esteem as a sexual partner. Dissatisfaction = dissatisfaction with sexual life. Preoccupation = preoccupation with sex.

**Table 2:** Logistic regression analyses of the use of Tinder.

	*b*	*SE*	OR	95% CI	*p*
Intercept	**−5.82**	**0.85**	**0.00**	**[0.00, 0.02]**	**<0.001**
Men	**0.42**	**0.19**	**1.52**	**[1.05, 2.19]**	**0.025**
Heterosexual	**−1.06**	**0.18**	**0.35**	**[0.24, 0.49]**	**<0.001**
Age	**0.22**	**0.04**	**1.25**	**[1.16, 1.35]**	**<0.001**

*SE* = standard error, OR = odds ratio, and CI = odds ratio confidence interval. Men: dummy variable where *women* = 0 and *men* = 1. Heterosexual: dummy variable where *sexual minority* = 0 and *heterosexual* = 1. Age, measured in years. Bold values correspond to statistically significant coefficients (*p* < 0.05).

**Table 3:** Multiple regression analysis of the Tinder use characteristics.

	**Usage Time**			**Use Frequency**			**# of People Met**		
Descriptives	*M*	*SD*	*Sk*	*M*	*SD*	*Sk*	*M*	*SD*	*Sk*
	4.04	6.08	2.68	10.14	7.41	0.36	2.59	3.89	2.87
Model	$R_{adj}^{2}$	*F*	*p*	$R_{adj}^{2}$	*F*	*p*	$R_{adj}^{2}$	*F*	*p*
	**0.06**	**4.65**	**0.004**	**0.03**	**3.01**	**0.032**	**0.04**	**3.22**	**0.024**
Coefficients	*b*	*SE*	*p*	*b*	*SE*	*p*	*b*	*SE*	*p*
Intercept	−6.63	4.74	0.164	−4.09	5.86	0.486	−3.27	3.07	0.288
Men	**2.14**	**0.99**	**0.032**	0.12	1.22	0.919	0.54	0.64	0.400
Heterosexual	−1.51	0.93	0.105	−2.11	1.14	0.067	**−** **1.33**	**0.60**	**0.029**
Age	**0.51**	**0.23**	**0.025**	**0.72**	**0.28**	**0.011**	**0.30**	**0.15**	**0.040**
	**# of Sexual Relations**			**# of Relationships**			**# of Friendships**		
Descriptives	*M*	*SD*	*Sk*	*M*	*SD*	*Sk*	*M*	*SD*	*Sk*
	1.32	2.82	4.51	0.27	0.48	1.46	0.85	1.65	4.60
Model	$R_{adj}^{2}$	*F*	*p*	$R_{adj}^{2}$	*F*	*p*	$R_{adj}^{2}$	*F*	*p*
	**0.03**	**2.91**	**0.036**	0.01	1.37	0.253	**0.10**	**7.18**	**<0.001**
Coefficients	*b*	*SE*	*p*	*b*	*SE*	*p*	*b*	*SE*	*p*
Intercept	−0.90	2.23	0.688	−0.12	0.39	0.750	−0.22	1.26	0.863
Men	0.62	0.46	0.187	−0.14	0.08	0.082	**0.70**	**0.26**	**0.008**
Heterosexual	**−** **0.98**	**0.44**	**0.026**	−0.06	0.08	0.411	**−** **0.81**	**0.25**	**0.001**
Age	0.12	0.11	0.251	0.02	0.02	0.230	0.06	0.06	0.298

*M* = mean. *SD* = standard deviation. *Sk* = skewness. *SE* = standard error; # = number. Usage time, measured in months. Use frequency, measured as times/week. Men: dummy variable where *women* = 0 and *men* = 1. Heterosexual: dummy variable where *sexual minority* = 0 and *heterosexual* = 1. Age, measured in years. Bold values correspond to statistically significant coefficients (*p* < 0.05).

**Table 4:** Multiple regression analysis of the Tinder motives scale.

	**Curiosity**			**Pastime**			**Sexual Orientation**			**Socializing**		
Descriptives	*M*	*SD*	*Sk*	*M*	*SD*	*Sk*	*M*	*SD*	*Sk*	*M*	*SD*	*Sk*
	4.83	1.42	−0.80	4.44	1.20	−0.64	4.15	1.87	−0.32	4.08	1.29	−0.35
Model	$R_{adj}^{2}$	*F*	*p*	$R_{adj}^{2}$	*F*	*p*	$R_{adj}^{2}$	*F*	*p*	$R_{adj}^{2}$	*F*	*p*
	**0.05**	**3.88**	**0.010**	**0.03**	**2.95**	**0.034**	**0.13**	**9.87**	**<0.001**	0.00	0.97	0.408
Coefficients	*b*	*SE*	*p*	*b*	*SE*	*p*	*b*	*SE*	*p*	*b*	*SE*	*p*
Intercept	0.37	0.79	0.638	−0.49	0.79	0.538	−0.87	0.75	0.250	−0.73	0.8	0.367
Men	**−** **0.53**	**0.16**	**0.001**	**−** **0.46**	**0.16**	**0.006**	0.14	0.16	0.371	−0.20	0.17	0.244
Heterosexual	0.04	0.15	0.797	−0.21	0.15	0.181	**−** **0.75**	**0.15**	**<0.001**	−0.18	0.16	0.255
Age	−0.01	0.04	0.772	0.04	0.04	0.354	0.06	0.04	0.099	0.04	0.04	0.278
	**Sexual Experience**			**Distraction**			**Relationship**			**Flirting/Social Skills**		
Descriptives	*M*	*SD*	*Sk*	*M*	*SD*	*Sk*	*M*	*SD*	*Sk*	*M*	*SD*	*Sk*
	3.45	1.53	−0.07	3.37	1.58	0.15	3.32	1.60	0.34	2.89	1.40	0.50
Model	$R_{adj}^{2}$	*F*	*p*	$R_{adj}^{2}$	*F*	*p*	$R_{adj}^{2}$	*F*	*p*	$R_{adj}^{2}$	*F*	*p*
	**0.03**	**2.87**	**0.038**	0.02	2.32	0.077	**0.03**	**2.95**	**0.034**	0.00	1.16	0.328
Coefficients	*b*	*SE*	*p*	*b*	*SE*	*p*	*b*	*SE*	*p*	*b*	*SE*	*p*
Intercept	−1.47	0.79	0.066	0.61	0.8	0.441	−2.22	0.79	0.006	−1.31	0.80	0.104
Men	0.27	0.16	0.101	**−** **0.38**	**0.17**	**0.023**	0.02	0.16	0.889	−0.10	0.17	0.562
Heterosexual	−0.19	0.15	0.215	−0.17	0.16	0.267	−0.16	0.15	0.310	0.09	0.16	0.560
Age	0.07	0.04	0.065	−0.02	0.04	0.624	**0.11**	**0.04**	**0.005**	0.06	0.04	0.118
	**Traveling**			**Social Approval**			**Peer Pressure**			**Ex**		
Descriptives	*M*	*SD*	*Sk*	*M*	*SD*	*Sk*	*M*	*SD*	*Sk*	*M*	*SD*	*Sk*
	2.54	1.66	0.94	2.53	1.28	0.43	2.20	1.42	1.22	2.17	1.63	1.41
Model	$R_{adj}^{2}$	*F*	*p*	$R_{adj}^{2}$	*F*	*p*	$R_{adj}^{2}$	*F*	*p*	$R_{adj}^{2}$	*F*	*p*
	**0.03**	**3.00**	**0.032**	0.01	1.57	0.199	**0.09**	**6.76**	**<0.001**	0.00	1.10	0.349
Coefficients	*b*	*SE*	*p*	*b*	*SE*	*p*	*b*	*SE*	*p*	*b*	*SE*	*p*
Intercept	−1.49	0.79	0.060	−1.58	0.8	0.051	−1.48	0.77	0.055	−0.51	0.80	0.527
Men	0.01	0.16	0.955	−0.15	0.17	0.358	**−** **0.47**	**0.16**	**0.004**	−0.29	0.17	0.083
Heterosexual	**−** **0.37**	**0.15**	**0.018**	−0.11	0.16	0.475	**0.36**	**0.15**	**0.017**	−0.02	0.16	0.917
Age	**0.08**	**0.04**	**0.035**	**0.08**	**0.04**	**0.040**	0.07	0.04	0.073	0.03	0.04	0.460
	**Belongingness**											
Descriptives	*M*	*SD*	*Sk*									
	1.66	0.96	1.67									
Model	$R_{adj}^{2}$	*F*	*p*									
	0.02	2.05	0.109									
Coefficients	*b*	*SE*	*p*									
Intercept	−1.15	0.8	0.151									
Men	**−** **0.35**	**0.17**	**0.037**									
Heterosexual	−0.01	0.16	0.958									
Age	0.06	0.04	0.122									

*M* = mean. *SD* = standard deviation. *Sk* = skewness. *SE* = standard error. Men: dummy variable where *women* = 0 and *men* = 1. Heterosexual: dummy variable where *sexual minority* = 0 and *heterosexual* = 1. Age, measured in years. Dependent variables were standardized. Motives were ordered by their means. Bold values correspond to statistically significant coefficients (*p* < 0.05).

**Table 5:** Multiple regression analysis of the different psychosexual variables.

	**PANAS Negative**			**PANAS Positive**			**MBSRQ**					
Descriptives	*M*	*SD*	*Sk*	*M*	*SD*	*Sk*	*M*	*SD*	*Sk*			
	21.96	6.83	0.67	34.64	6.44	−0.48	24.97	6.37	−0.58			
Model	$R_{adj}^{2}$	*F*	*p*	$R_{adj}^{2}$	*F*	*p*	$R_{adj}^{2}$	*F*	*p*			
	**0.02**	**5.81**	**<0.001**	**0.02**	**7.94**	**<0.001**	**0.02**	**7.63**	**<0.001**			
Coefficients	*b*	*SE*	*p*	*b*	*SE*	*p*	*b*	*SE*	*p*			
Intercept	0.66	0.29	0.023	**−1.34**	**0.29**	**<0.001**	**−1.50**	**0.29**	**<0.001**			
Men	0.01	0.07	0.869	**−0.15**	**0.07**	**0.021**	−0.07	0.07	0.294			
Heterosexual	**−0.27**	**0.07**	**<.001**	0.07	0.07	0.286	0.13	0.07	0.054			
Age	−0.02	0.01	0.102	**0.06**	**0.01**	**<0.001**	**0.07**	**0.01**	**<0.001**			
Tinder used	0.12	0.08	0.146	0.13	0.08	0.113	−0.08	0.08	0.346			
	**SSS Sexual Partner**			**SSS Dissatisfaction**			**SSS Preoccupation**					
Descriptives	*M*	*SD*	*Sk*	*M*	*SD*	*Sk*	*M*	*SD*	*Sk*			
	18.87	4.04	–0.47	9.91	5.18	1.05	8.75	4.30	1.39			
Model	$R_{adj}^{2}$	*F*	*p*	$R_{adj}^{2}$	*F*	*p*	$R_{adj}^{2}$	*F*	*p*			
	**0.01**	**5.14**	**<0.001**	**0.05**	**18.15**	**<0.001**	**0.05**	**17.38**	**<0.001**			
Coefficients	*b*	*SE*	*p*	*b*	*SE*	*p*	*b*	*SE*	*p*			
Intercept	**−1.05**	**0.29**	**<0.001**	0.83	0.29	0.003	0.39	0.29	0.171			
Men	**−0.15**	**0.07**	**0.030**	**0.44**	**0.07**	**<.001**	**0.42**	**0.07**	**<0.001**			
Heterosexual	0.02	0.07	0.793	−0.10	0.07	0.119	−0.04	0.07	0.570			
Age	**0.05**	**0.01**	**<0.001**	**−0.04**	**0.01**	**0.001**	−0.02	0.01	0.071			
Tinder used	0.09	0.08	0.300	**0.28**	**0.08**	**<0.001**	**0.37**	**0.08**	**<0.001**			
	**SOI–R Behavior**			**SOI–R Attitude**			**SOI–R Desire**			**CNAS**		
Descriptives	*M*	*SD*	*Sk*	*M*	*SD*	*Sk*	*M*	*SD*	*Sk*	*M*	*SD*	*Sk*
	8.48	5.76	1.29	17.88	6.75	–0.46	10.26	5.48	0.70	29.97	10.40	0.17
Model	$R_{adj}^{2}$	*F*	*p*	$R_{adj}^{2}$	*F*	*p*	$R_{adj}^{2}$	*F*	*p*	$R_{adj}^{2}$	*F*	*p*
	**0.15**	**57.5**	**<0.001**	**0.06**	**19.58**	**<0.001**	**0.14**	**50.26**	**<0.001**	**0.13**	**49.46**	**<0.001**
Coefficients	*b*	*SE*	*p*	*b*	*SE*	*p*	*b*	*SE*	*p*	*b*	*SE*	*p*
Intercept	**−2.55**	**0.27**	**<0.001**	−0.09	0.28	0.744	**0.61**	**0.27**	**0.024**	**0.97**	**0.27**	**<0.001**
Men	**−0.19**	**0.06**	**0.003**	0.06	0.07	0.344	**0.56**	**0.06**	**<0.001**	−0.04	0.06	0.499
Heterosexual	**−0.20**	**0.06**	**0.002**	**−0.45**	**0.07**	**<.001**	**−0.42**	**0.06**	**<0.001**	**−0.83**	**0.06**	**<0.001**
Age	**0.13**	**0.01**	**<0.001**	0.02	0.01	0.167	−0.02	0.01	0.069	−0.02	0.01	0.202
Tinder used	**0.65**	**0.08**	**<0.001**	**0.31**	**0.08**	**<.001**	**0.52**	**0.08**	**<0.001**	**0.22**	**0.08**	**0.005**

*M* = mean. *SD* = standard deviation. *Sk* = skewness. *SE* = standard error. Men: dummy variable where *women* = 0 and *men* = 1. Heterosexual: dummy variable where *sexual minority* = 0 and *heterosexual* = 1. Tinder used: dummy variable where *no* = 0 and *yes* = 1. Age, measured in years. Dependent variables were standardized. Bold values correspond to statistically significant coefficients (*p* < 0.05). PANAS = Positive and Negative Affect Schedule. MBSRQ = Appearance Evaluation Scale of the Multidimensional Body-Self Relations Questionnaire-Appearance Scales. SSS = Short version of the Sexuality Scale. SOI-R = Sociosexual Orientation Inventory-Revised. CNAS = Consensual Nonmonogamy Attitude Scale. Sexual Partner = self-esteem as a sexual partner. Dissatisfaction = dissatisfaction with sexual life. Preoccupation = preoccupation with sex.

## Appendix A

**Table A1:** Spanish version of the Tinder Motives Scale [22].

Please, answer honestly on a scale from “Strongly disagree” to “Strongly agree”. I use Tinder...	Responda sinceramente en una escala que va de “Totalmente en desacuerdo” a “Totalmente de acuerdo”. Uso Tinder...
1. As a break at work or during a study period.	1. Como descanso durante el trabajo o estudio.
2. To live out a sexual fantasy.	2. Para vivir una fantasía sexual.
3. To gain more self-confidence in my social skills.	3. Para tener más confianza en mis relaciones sociales.
4. Because I want to be trendy.	4. Porque quiero estar a la moda.
5. For fun.	5. Por diversión.
6. To seek out someone to date.	6. Para buscar alguien con quien tener una cita.
7. Because it passes time when I´m bored.	7. Porque me entretiene cuando estoy aburrido.
8. When I have nothing better to do.	8. Cuando no tengo nada mejor que hacer.
9. Because it is a fad.	9. Porque es una moda.
10. So that I do not focus my attention on my ex anymore.	10. Para no prestarle atención a mi ex nunca más.
11. To see how desirable I am.	11. Para comprobar lo deseable que soy.
12. To build and emotional connection with someone.	12. Para conseguir una conexión emocional con otra persona.
13. To meet a future husband or wife.	13. Para encontrar un futuro esposo/esposa.
14. Because it is entertaining.	14. Porque es entretenido.
15. To find a lover/mistress.	15. Para encontrar un/a amante/persona con quien tener sexo.
16. To procrastinate things I should be doing (working, studying, etc.).	16. Para posponer lo que debería estar haciendo (trabajar, estudiar, etc.).
17. To see what the application is about.	17. Para ver de qué trata la aplicación.
18. To think less about my ex.	18. Para pensar menos en mi ex.
19. To broaden my social network.	19. Para ampliar mi red de conocidos/as.
20. To increase my sexual experience.	20. Para tener más experiencia sexual.
21. To meet new people.	21. Para conocer gente nueva.
22. Because everyone uses Tinder.	22. Porque todo el mundo usa Tinder.
23. To fall in love.	23. Para enamorarme.
24. Because it is a more enjoyable to make the first move.	24. Porque es más excitante dar el primer paso.
25. To be able to better estimate my own attractiveness	25. Para estimar mejor mi propio atractivo.
26. To pass time.	26. Para pasar el rato.
27. To find a one-night stand.	27. Para encontrar un rollo de una noche.
28. To get an "ego-boost”.	28. Para aumentar mi ego.
29. To find a friend-with-benefits/fuckbuddy.	29. Para encontrar un/a amigo/acon beneficios/follamigo.
30. To learn about hotspots in foreign countries through locals.	30. Para aprender de los autóctonos sobre los sitios más populares en países extranjeros.
31. To relax.	31. Para relajarme.
32. To see how easy it is to find a sex partner.	32. Para comprobar lo fácil que es encontrar una pareja sexual.
33. Out of curiosity.	33. Por curiosidad.
34. To combat boredom when working or studying.	34. Para combatir el aburrimiento mientras trabajo o estudio.
35. To connect with other people with the same sexual orientation.	35. Para contactar con gente con la misma orientación sexual que yo.
36. To increase my flirting experience.	36. Para aumentar mi experiencia en ligues/coqueteos.
37. To make new friends.	37. Para hacer nuevos amigos.
38. To get to know people with the same sexual orientation.	38. Para llegar a conocer gente con la misma orientación sexual que yo.
39. To occupy my time.	39. Para estar ocupado/a.
40. To easily find people that are willing to party when in a foreign country.	40. Para encontrar fácilmente gente que quiera salir de fiesta cuando estoy en un país extranjero.
41. Because my friends thought I should use Tinder.	41. Porque mis amigos piensan que debería usar Tinder.
42. To improve my social skills.	42. Para mejorar mis habilidades sociales.
43. To try it out.	43. Para probarlo.
44. Because someone else made me a Tinder profile.	44. Porque alguien me creó un perfil en Tinder.
45. To learn to flirt.	45. Para aprender a ligar/coquetear.
46. To find someone for a serious relationship.	46. Para encontrar a alguien para tener una relación seria.
47. To broaden my social network when on an abroad/exchange experience.	47. Para aumentar mi red de conocidos cuando estoy de viaje.
48. To meet singles with a similar sexual orientation.	48. Para conocer solteros/as con la misma orientación sexual que yo.
49. To get tips from locals (in restaurants, shopping, parties, etc.) when travelling.	49. Para recibir consejos de los autóctonos (sobre restaurantes, tiendas, fiestas, etc.) cuando viajo.
50. To meet other travelers/locals when in a foreign country.	50. Para conocer a otros turistas/autóctonos cuando estoy en un país extranero.
51. To get attention.	51. Para llamar la atención.
52. To get compliments.	52. Para recibir cumplidos.
53. To talk to people I don´t know personally.	53. Para hablar con alguien a quien no conozco personalmente.
54. As suggested by friends.	54. Sugerido por mis amigos.
55. To get over my ex.	55. Para olvidar a mi ex.
56. To be cool.	56. Para ser guay/cool.
57. Because it is hard to talk to people in real life.	57. Porque es complicado hablar con alguien en la vida real.
58. To get self-validation from others.	58. Para conseguir la aprobación de los demás.

1 = Totalmente en desacuerdo/2 = En desacuerdo/3 = Más bien en desacuerdo/4 = Ni de acuerdo ni en desacuerdo/5 = Más bien de acuerdo/6 = De acuerdo/7 = Totalmente de acuerdo.

## Appendix B

**Table A2:** Spanish version of the Consensual Nonmonogamy Attitude Scale [39].

Please rate the extent to which you agree with the following statements:	Por favor, señale en qué medida está de acuerdo con las siguientes afirmaciones:
1. You must be in a monogamous relationship to be in love.	1. Una persona debe estar en una relación monógama para estar enamorada.
2. I can see myself entering into a non-monogamous relationship.	2. Puedo imaginarme a mí mismo teniendo una relación no monógama.
3. A monogamous relationship is the most satisfying type of relationship.	3. Una relación monógama es el tipo de relación más satisfactorio.
4. Intimate relationships with more than one person are too complicated.	4. Tener relaciones íntimas con más de una persona es demasiado complicado.
5. It is possible to have several satisfying intimate relationships at the same time.	5. Es posible tener varias relaciones íntimas satisfactorias al mismo tiempo.
6. It is possible to date other people while in a love relationship with your partner.	6. Es posible salir con otras personas mientras tienes una relación romántica con tu pareja.
7. It is possible to have sexual relationships with other people while in a loving relationship with your partner.	7. Es posible tener relaciones sexuales con otras personas mientras tienes una relación romántica con tu pareja.
8. It is possible for one partner in a relationship to be monogamous while the other partner is not monogamous.	8. Es posible que un miembro de una pareja sea monógamo mientras que el otro no lo sea.

1 = Totalmente en desacuerdo/2/3/4 = Neutral/Ni de acuerdo ni en desacuerdo/5/6/7 = Totalmente de acuerdo.
